# A randomized non-inferiority study of low-dose and standard-dose ticagrelor after intervention for acute coronary syndrome: study protocol for the TIGER STUDY

**DOI:** 10.1186/s13063-022-06124-z

**Published:** 2022-03-05

**Authors:** Yanan Pang, Minglu Ma, Jiachun Xia, Dong Wang, YanChen, Jianfei Ye, Zhongwei Jia, Sicheng Wu, Jian Yang, Lei Hou

**Affiliations:** 1 The Department of Cardiology, Institute of Cardiovascular Diseases, Tongren Hospital, Shanghai Jiao Tong University School of Medicine, 200336 Shanghai, China; 2 The Department of Cardiology, Taishan People’s Hospital, 529000 Guangdong, China; 3 The Department of Cardiology, Ningbo Fourth Hospital, 315700 Zhejiang, China; 4 The Department of Cardiology, Southwestern Lu hospital, 252300 Shandong, China; 5 Biostatistics Office of Clinical Research Center, Shanghai Ninth People’s Hospital, Shanghai Jiao Tong University School of Medicine, 200011 Shanghai, China; 6 The Department of Cardiology, Yanzhou Branch of Affiliated Hospital of Jining Medical University, 272199 Shandong, China

**Keywords:** Acute coronary syndrome, Intervention, Ticagrelor, Dual antiplatelet therapy

## Abstract

**Background:**

Current guidelines recommend that patients with acute coronary syndrome (ACS) who have successfully undergone percutaneous coronary intervention (PCI) should continue to use dual antiplatelet therapy (DAPT) for 12 months. The long-term use of standard-dose dual antiplatelet therapy will increase the risk of bleeding. An optimized antiplatelet strategy that can prevent ischemic events and reduce the risk of bleeding remains to be explored.

**Methods:**

The study is a prospective, multicenter, randomized, open-label, controlled study involving 2090 patients from six clinical centers in China. Through the interactive web response system (IWRS), ACS patients undergoing successful PCI will be randomly divided into the low-dose ticagrelor group or the normal-dose ticagrelor group, after taking 100 mg aspirin and 90 mg ticagrelor bid for 1 week. The primary endpoint is a composite of cardiovascular death, non-fatal myocardial infarction, stent thrombosis, repeat revascularization, and stroke. The secondary endpoints are bleeding events of grade 2 or higher according to Bleeding Academic Research Consortium [BARC] criteria, cardiovascular death, acute myocardium infarction, and coronary revascularization at 1 year.

**Discussion:**

Recent studies have confirmed that 90 mg ticagrelor alone can safely and effectively reduce bleeding without increasing ischemic events of patients with ACS after PCI. Compared with standard-dose DAPT, whether low-dose ticagrelor combined with aspirin can ensure the anti-ischemic effect while reducing the bleeding risk remains unclear in Chinese patients.

The TIGER study will be the first large-scale, multicenter study to compare the efficacy and safety of low-dose and standard-dose ticagrelor combined with aspirin in ACS patients 1 week after successful PCI.

**Trial registration:**

Clinicaltrials.gov NCT04255602. Registered on 5 February 2020.

## Background

Recent guidelines recommended 12-month dual antiplatelet therapy (DAPT) with aspirin and P2Y12 receptor antagonist ticagrelor for ACS patients who undergo PCI with drug-eluting stents (DES) [1, 2]. However, standard-dose DAPT is companied with the incidence of bleeding events to 3–10% [3]. Gastrointestinal hemorrhage is even an independent risk factor for death in ACS patients [4, 5]. The optimized antiplatelet strategy, which could inhibit ischemic events while reducing bleeding events versus standard-dose DAPT, is of vital importance.

Recent clinical trials, such as TWILIGHT and Global Leaders have confirmed that 90 mg ticagrelor alone can safely and effectively reduce bleeding in patients with ACS after PCI [6–8]. Moreover, SMART-CHOICE trial suggested that P2Y12 monotherapy may be a better choice for the Asian ACS population [9]. These studies challenge the current DAPT strategy. However, aspirin should not be given up easily, considering that ticagrelor has a higher rate of discontinuation, the increased frequency of dyspnea, and higher costs than aspirin. Therefore, the optimal DAPT strategy still needs to be explored.

The TIFU study indicated that the low-dose dual antiplatelet drugs may be another choice for ACS patients [10]. PEGASUS-TIMI 54 study and the latest ELECTRA study showed 60 mg ticagrelor has same platelet inhibitory effect as that of 90 mg ticagrelor in patients undergoing PCI [11, 12]. However, larger-scale and high-quality clinical controlled studies are required to confirm these findings.

The TIGER study decided to compare the safety and efficacy of low-dose DAPT (ticagrelor 60 mg bid plus aspirin 100 mg qd) and standard-dose DAPT (ticagrelor 90 mg bid plus aspirin 100 mg qd) following 1-week standard-dose DAPT in ACS patients after successful PCI, aiming to explore the optimal DAPT strategy for ACS patients.

## Methods

### Study objectives and hypothesis

The main purpose of this study is to compare the safety and efficacy of low-dose and standard-dose ticagrelor with aspirin in ACS patients 1 week after successful PCI. We hypothesized that low-dose ticagrelor group would be non-inferior to the standard-dose ticagrelor group in preventing ischemic events while reducing the risk of bleeding.

### Study design and setting

The TIGER study is a prospective, multicenter, randomized, open-label, controlled clinical trial, involving 2090 participants from six heart clinical centers in China. All ACS patients who meet all the enrollment criteria will be randomly assigned to one of the two groups 1 week after the successful PCI: low-dose ticagrelor group (ticagrelor 60 mg + ASA 100 mg; *n* = 1045) and standard-dose ticagrelor group (ticagrelor 90 mg + ASA 100 mg; *n* = 1045), using the interactive web response system (IWRS), at a 1:1 ratio.

All patients will receive standard DAPT (aspirin 100 mg qd + ticagrelor 90 mg bid) therapy in the first week after PCI. Then, patients will take different dose ticagrelor combined with aspirin according to their group within 1 year after surgery. To ensure drug compliance, telephone call will be done monthly by specific staff.

If patients are intolerance to ticagrelor or have ischemic or bleeding complications during the first week after PCI, he or she will be recorded as drop-out and will receive timely and carefully therapy according the exactly situations.

**Fig. 1:**
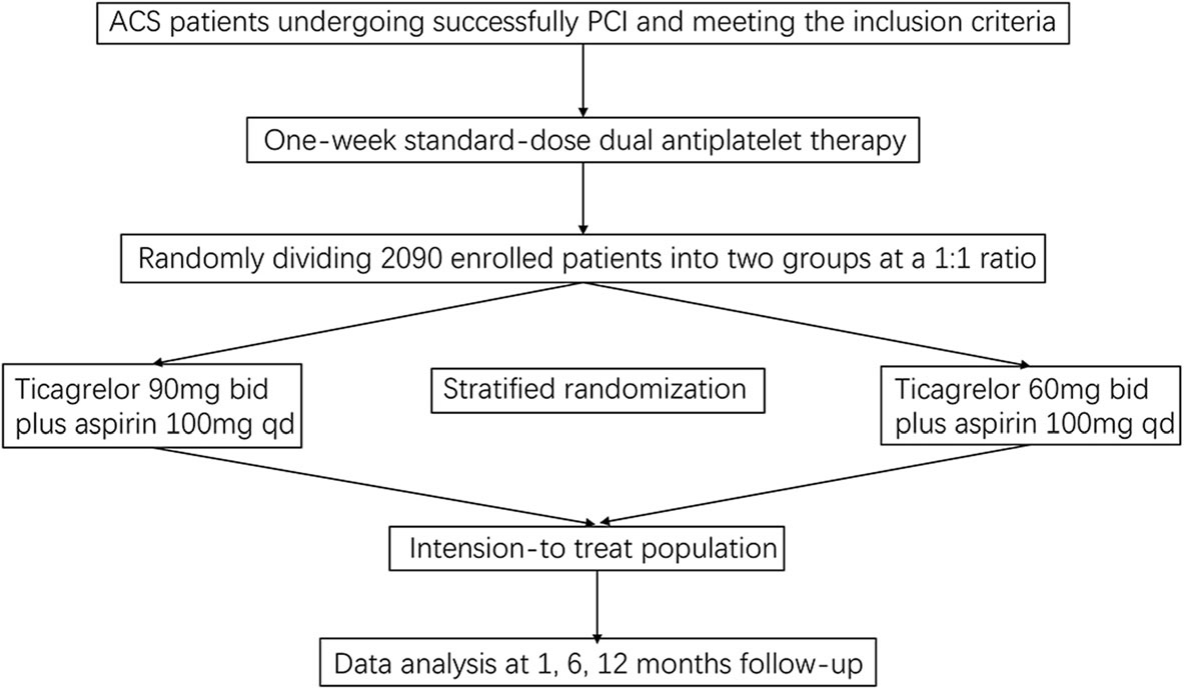
Inclusion flow chart

Clinical follow-up will be performed by telephone or office visit at 1 month, 6 months, and 12 months after PCI. The research flow chart is shown in Fig. 1. The follow-up schedule is shown in Table 1.

**Table 1:** Follow-up schedule

Follow-up time point (after PCI)	± Days
1 month	20
6 months	20
12 months	20

### Randomization

Our study will strictly follow the principle of randomization. After signing of the informed consent, using the interactive web response system (IWRS) by physician, enrolled patients will be randomly divided into low-dose ticagrelor group (*n* = 1045) and standard-dose ticagrelor group (*n* = 1045). The randomization will be stratified by ACS (STEMI/non-STEMI/UAP), age (< 75/≥75), sex (male/female), and diabetes (yes/no).

### Study population and enrollment criteria

In the TIGER study, a total of 2090 ACS patients receiving successful PCI and 1-week standard-dose DAPT will be enrolled in six cardiac clinical centers. Acute coronary syndrome (ACS) includes ST-elevation myocardial infarction, non-ST-elevation myocardial infarction, and unstable angina [13]. It can be confirmed by clinical symptoms and relevant medical examination. Patients recruited must meet all inclusion criteria and without any exclusion criteria. Patients over 18 years old and less than 90 years old who were diagnosed with ACS and successfully received PCI are eligible for inclusion. The informed consent must be signed before they enter the study. Patients who are allergic to aspirin or ticagrelor cannot participate in the study. Since our study will last 2 years, patients with life expectancy less than 2 years need to be excluded. Meanwhile, considering the impact of pregnancy on individual physiology, pregnant women or women who are going to be pregnant in the next 2 years will not be recruited. Patients with a history of cerebral hemorrhage, stroke within half a year, active hemorrhage, or known hemorrhage diseases will be excluded because of the great risk of bleeding. In order to ensure the compliance and safety of patients, patients with the following conditions should also be excluded, such as several liver and kidney disorders (ALT > 5 times ULA, EGFR < 15 ml/min/1.73 mm^2^), malignant tumor diseases, platelets less than 100 × 10^9^/L or hemoglobin less than 90 g/L, requiring oral anticoagulants, and patients considered unsuitable for this study by the researchers [14, 15]. In all cases, the investigator based on clinical factors and a review of the initial angiogram will make the final decision whether or not to recruit the patient. All the inclusion and exclusion criteria details are described in Table 2.

**Table 2:**
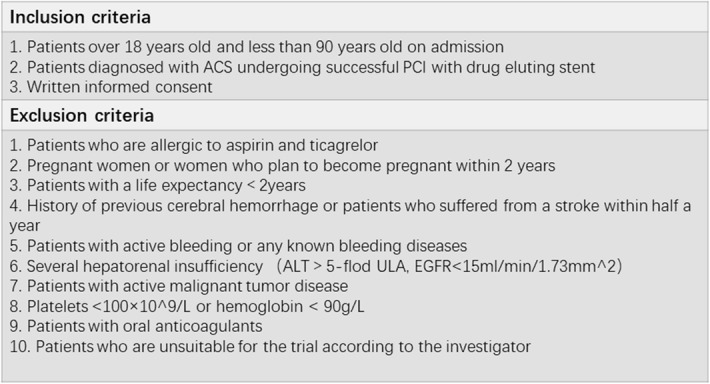
Enrollment criteria

### Interventions

Antiplatelet drugs are needed to prevent stent thrombosis before and after PCI with DES in ACS patients. If the ACS patient is not taking aspirin before, 300 mg loading dose aspirin must be taken at least 24 h before PCI. Aspirin will then continue to be administered at a dose of 100 mg once daily until 1 year after intervention. Similarly, if the patient did not take ticagrelor, they should use 180 mg loading dose ticagrelor at least 2 h before PCI. Even if patients cannot get ticagrelor 2 h before operation, they will get it in the cardiac catheterization room before it. Within 1 week after the operation, oral administration of ticagrelor 90 mg twice daily should be continuously applied in both groups. Then, patients will be given the corresponding dose ticagrelor (90 mg bid or 60 mg bid) according to the group they entered. The use of aspirin and ticagrelor will last for 1 year after PCI. Notably, additional antiplatelet drugs will not be allowed to use during the period of the study. In our study, only second-generation drug-eluting stents will be used.

### Outcomes

The primary endpoint is the composite endpoint of cardiovascular death, non-fatal myocardial infarction, stent thrombosis, revascularization, and stroke within 1 year after PCI [16]. The secondary endpoints are bleeding events of grade 2 or higher according to Bleeding Academic Research Consortium [BARC] criteria, cardiovascular death, acute myocardium infarction, and coronary revascularization at 1 year [16]. All clinical outcomes are defined according to the Academic Research Consortium (ARC) [17]. All adverse events will be reported to the ethics committee (EC) in a given time. The endpoints will be adjudicated by the Clinical Event Adjudication Committee (CEAC).

### Sample size calculation

In our study, we assume that the composite of MACCE (defined as a composite of all-cause death, myocardial infarction, or stroke at 12 months after PCI) in the control group will be 5% at 1 year in ACS after PCI according to previous studies (STOD-DAPT2, RESET) [18, 19] The inferiority margin was set to 2.5% with clinical considerations. The sample size of the study should not be smaller than 1880 (940 per group) to guarantee 80% power of the study at one-sided significance level of 0.05. Considering 10% of the patients would be lost in the follow-up period, we determined the final sample size to be 2090 patients (1045 per group).

### Statistical analysis

The efficacy analysis for primary endpoint will be performed in the intent-to-treat population, safety endpoints analyses will be performed in the safety analysis population.

The primary endpoint between groups will be compared with the use of logistic regression, adjusted for the stratification factors (ACS, age, sex, and diabetes). Odds ratio (OR) and the corresponding confidence interval (CI) will be estimated. If the upper limit of the two-sided 90% CI of the OR was less than the prespecified non-inferiority margin, low-dose DAPT strategy will be considered non-inferior to standard DAPT strategy in 1-year period after PCI in ACS patients. Besides, cumulative event rates will be estimated with the Kaplan-Meier method. Patients who were lost to follow-up were censored at the time of the last known contact.

Continuous variable will be descripted as mean (std) or median (interquartile range) as appropriate; categorical variables will be descripted as counts and percentages. *P* values and CIs were 2-tailed except those for non-inferiority testing of the primary end point.

No interim efficacy analysis is planned in this study, an independent data monitor committee (IDMC) will be set up to assess the safety of subjects periodically as previously described.

### Ethical considerations

The researchers must obtain the approval of the study protocol and informed consent from the ethics committee. Meanwhile, researchers will be responsible for regular reporting as required by the institutional committee over the whole study period.

Any protocol amendments as well as associated informed consent changes will be submitted to ethics committee and written approval must be obtained prior to implementation. The informed consent form should be resigned when any modifications to the protocol happened.

Regarding the enrolled patients, we must obtain the informed consent signed by them in advance. The privacy of patients will be kept in strictly confidential during and after the TIGER study. All questions of privacy and secrecy are listed in the informed consent form (ICF). Enrolled patients have the right to withdraw from the trial anytime during the process of the study. All data will only be accessed by the study staff and kept strictly confidential. Patient data will be protected by locked cabinets at the Clinical Centers, and use of passwords limited access storage of electronic data.

Independent data monitor committee (IDMC) will be notified of all serious adverse events and unanticipated adverse device events occurring during the study. IDMC will also review compiled adverse event data at periodic intervals and report to the ethics committee any safety concerns and recommendations for suspension or early termination of the trial.

### Study organization

The TIGER study is a multicenter, prospective, randomized, open-label, controlled study designed and initiated by Tongren Hospital affiliated to Shanghai Jiaotong University Medical College. Doctor Lei Hou is the principal investigator. We guarantee to obtain the informed consent and approval of the ethics committee before starting any research procedure. This study will compare the safety and efficacy of low- and standard- dose ticagrelor combined with aspirin in ACS patients 1 week after undergoing successful PCI with DES. The executive committee will be responsible for the scientific operational of the study. The independent data monitoring committee (IDMC) will be responsible for reviewing the data on a regular basis and determining the safety scope of the test, as well as the termination of the test, and the executive committee will finally decide whether to terminate the study ahead of time based on the recommendation of IDMC. The study will fully follow the ethical principles of the Helsinki declaration.

There is a sponsor that is separate from the funder and the sponsor and funder are independent. All data will be collected in case report forms (CRFs) and will be entered into electronic record form by two independent researchers to ensure the accuracy of the data. The data collectors will be trained to fill out the CRFs to ensure the accuracy of the data. Study staff will keep in touch with the participants every month by calls and encourage participants to see specialized research clinics when they have any concerns about their health conditions or the study. All data will be collected in case report forms (CRFs) and will be entered into electronic record form by two independent researchers to ensure the accuracy of the data. The missing data will be filled using the last observation carry-over method. We plan to publish the trial results in peer reviewed journal. Readers who want to check the original data can contact the corresponding author.

## Discussion

The introduction of second-generation drug-eluting stents and optimal medical therapy has significantly decreased the incidence of stent thromboses. Bleeding has gradually emerged as a predictor of early and late mortality in patients with ACS [3]. Therefore, avoiding bleeding events has become essential after using antiplatelet drugs for ACS patients undergoing PCI.

Previous studies have shown the advantage of short period of DAPT strategy in preventing ischemic events and reducing the risk of bleeding [7, 9, 18]. However, ACS patients usually present with concomitant diseases such as ischemic stroke and transient ischemic attack in which aspirin is essential for the secondary prevention. On the other hand, some small-scale studies showed that ticagrelor 60 mg bid achieved high peak levels through platelet inhibition in nearly all patients, similar to that of ticagrelor 90 mg bid [11, 20].

Therefore, we designed the TIGER study to compare the safety and efficacy of low-dose and standard-dose ticagrelor combined with aspirin in patients suffering from ACS after successful PCI. We believe the TIGER study will offer a good choice to find an optimal antiplatelet strategy for ACS patients after PCI.

## Trial status

Recruitment started on February, 2020, and is planned to end in February, 2022, with 2090 patients randomized. The current protocol version is 1.1, dated February 1, 2020.

## Data Availability

Not applicable.

## Abbreviations

ACS: Acute coronary syndrome
DAPT: Dual antiplatelet therapy
PCI: Percutaneous coronary intervention
DES: Drug eluting stent
IWRS: Interactive web response system
IDMC: Independent data monitor committee
CEAC: Clinical Event Adjudication Committee
